# Lactoferrin selectively triggers apoptosis in highly metastatic breast cancer cells through inhibition of plasmalemmal V-H+-ATPase

**DOI:** 10.18632/oncotarget.11394

**Published:** 2016-08-19

**Authors:** Cátia S. Pereira, Joana P. Guedes, Marília Gonçalves, Luís Loureiro, Lisandra Castro, Hernâni Gerós, Lígia R. Rodrigues, Manuela Côrte-Real

**Affiliations:** ^1^ Centre of Molecular and Environmental Biology (CBMA), Department of Biology, University of Minho, Braga, Portugal; ^2^ Centre of Biological Engineering (CEB), Department of Biological Engineering, University of Minho, Braga, Portugal; ^3^ Centre for the Research and Technology of Agro-Environmental and Biological Sciences (CITAB), Department of Biology, University of Minho, Braga, Portugal

**Keywords:** lactoferrin, V-H^+^-ATPase, breast cancer, V-H^+^-ATPase inhibitor, extracellular acidification rate

## Abstract

Breast cancer is the most common type of cancer affecting women. Despite the good prognosis when detected early, significant challenges remain in the treatment of metastatic breast cancer. The recruitment of the vacuolar H^+^-ATPase (V-H^+^-ATPase) to the plasma membrane, where it mediates the acidification of the tumor microenvironment (TME), is a recognized feature involved in the acquisition of a metastatic phenotype in breast cancer. Therefore, inhibitors of this pump have emerged as promising anticancer drugs. Lactoferrin (Lf) is a natural pro-apoptotic iron-binding glycoprotein with strong anticancer activity whose mechanism of action is not fully understood. Here, we show that bovine Lf (bLf) preferentially induces apoptosis in the highly metastatic breast cancer cell lines Hs 578T and MDA-MB-231, which display a prominent localisation of V-H^+^-ATPase at the plasma membrane, but not in the lowly metastatic T-47D or in the non-tumorigenic MCF-10-2A cell lines. We also demonstrate that bLf decreases the extracellular acidification rate and causes intracellular acidification in metastatic breast cancer cells and, much like the well-known proton pump inhibitors concanamycin A and bafilomycin A1, inhibits V-H^+^-ATPase in sub-cellular fractions. These data further support that bLf targets V-H^+^-ATPase and explain the selectivity of bLf for cancer cells, especially for highly metastatic breast cancer cells. Altogether, our results pave the way for more rational *in vivo* studies aiming to explore this natural non-toxic compound for metastatic breast cancer therapy.

## INTRODUCTION

Despite the therapeutic advances of the last century, breast cancer is still the most common cancer in women worldwide. In this context, an efficient diagnostic and treatment of breast cancer remains a huge challenge due to its heterogeneity and metastasis incidence [1, 2]. Therefore, the search for new targets and drugs to improve breast cancer therapy is of great importance.

The vacuolar H^+^-ATPase (V-H^+^-ATPase), which actively transports protons across cellular membranes of eukaryotic cells, has been suggested as an attractive target for breast cancer therapy [3]. This notion is supported by different studies with various breast cancer cell lines, which implicated V-H^+^-ATPase in breast cancer survival, pH homeostasis, invasiveness, and in the acquisition of a more metastatic phenotype [4–7]. In addition, V-H^+^-ATPase was shown to display higher activity and to localise at the plasma membrane of highly metastatic breast cancer cells in comparison with poorly metastatic cells and immortalized breast cells [4, 7]. Accordingly, highly metastatic breast cancer cells were found to be more sensitive to V-H^+^-ATPase inhibitors than poorly metastatic cells, which were shown to preferentially use the Na^+^/H^+^ exchanger over V-H^+^-ATPase to acidify the extracellular medium [7].

Given the contribution of V-H^+^-ATPase to the acidity of the tumour microenvironment (TME) and its recognized importance in breast cancer, inhibitors of this pump have emerged as excellent candidates for breast cancer therapy. However, the most well-known V-H^+^-ATPase inhibitors concanamycin A (ConcA) and bafilomycin A1 (BafA1) were found to be extremely toxic *in vivo* and therefore not suitable for clinical use [8, 9]. Meanwhile, new V-H^+^-ATPase inhibitor classes have emerged such as benzolactone enamide salicylihalamide [10], indole derivatives [11], macrolacton archazolids [12], among others (reviewed by [13]). These compounds have been reported as exhibiting increased selectivity to cancer cells when compared to the classical ConcA and BafA1 inhibitors. In fact, the cytotoxic effect of archazolid B was shown to be much more prominent in cancer cells than in non-cancer cells derived from breast, kidney and umbilical vein [12]. However, the clinical exploitation of these compounds is far from being attainable.

Lactoferrin (Lf) is a natural iron-binding glycoprotein present in many tissues and biological fluids, such as milk, which is produced by mucosal epithelial cells or neutrophils during inflammation processes [14]. Among the many different biological activities assigned to Lf, its anticancer activity has been observed in different cell lines, animal models and even in clinical trials. Indeed, many *in vitro* and *in vivo* studies indicate that the anticancer activity of this protein is related with its capacity to induce apoptosis and to modulate the levels of key apoptotic molecules. Particularly, Lf was shown to downregulate the levels of the anti-apoptotic protein Bcl-2 [15–18], to increase the expression of the pro-apoptotic Bax protein [15, 16], to activate caspase-3 [15, 19, 20], caspase-9 [21] and caspase-8, to promote poly(ADP-ribose) polymerase (PARP) cleavage [19, 21], to increase Fas expression [19], and to activate p53 [22]. Also, in a large scale proteomic analysis of breast cancer cells, 9% of the proteins upregulated by Lf were classified as involved in apoptosis [23]. In another study with breast cancer cells, key apoptotic molecules modulated by Lf were identified using a human apoptosis protein array, namely p53, Bcl-2 family proteins, inhibitors of apoptosis proteins (IAPs) members, like survivin, and their inhibitors [24]. Since Lf is a non-toxic and low-cost dietary protein with a strong anticancer activity, it has a potential widespread application in cancer therapy. However, the anticancer mechanism of Lf is not fully understood, which limits its exploitation in the clinic [25].

Given the effectiveness of Lf against breast cancer cell lines [16, 18, 24, 26] and the aforementioned importance of V-H^+^-ATPase in breast cancer, we hypothesized that Lf could act as a V-H^+^-ATPase inhibitor in these cancer cells. To address our hypothesis, we assessed the effect of bovine Lf (bLf) on cell proliferation, apoptosis induction, extracellular acidification rate, intracellular pH, as well as the localisation of V-H^+^-ATPase in three different breast cell lines, namely the highly metastatic cancer cell line Hs 578T, the poorly metastatic cancer cell line T-47D, and the non-tumorigenic cell line MCF-10-2A. The induction of apoptosis by bLf and its relation with intracellular acidification was also addressed in the highly metastatic breast cancer cell line MDA-MB-231 to further support that this protein is preferentially cytotoxicity against highly metastatic cancer cells. Also, the effect of bLf on the biochemical activity of V-H^+^-ATPase was evaluated in lysosomes isolated from rat liver and crude membrane fractions from a cancer cell line, and compared with the inhibitory effect of ConcA and BafA1.

A step towards the bLf mechanism of action was crossed in this study since we identified for the first time V-H^+^-ATPase as a molecular target of bLf, which underlies its selectivity for highly metastatic breast cancer cells. Thus, we propose this protein as a new V-H^+^-ATPase inhibitor with promising therapeutic applications in breast cancer.

## RESULTS

### The susceptibility of breast cell lines to bovine lactoferrin is associated with a differential extracellular acidification rate and V-H^+^-ATPase localisation

**Figure 1:**
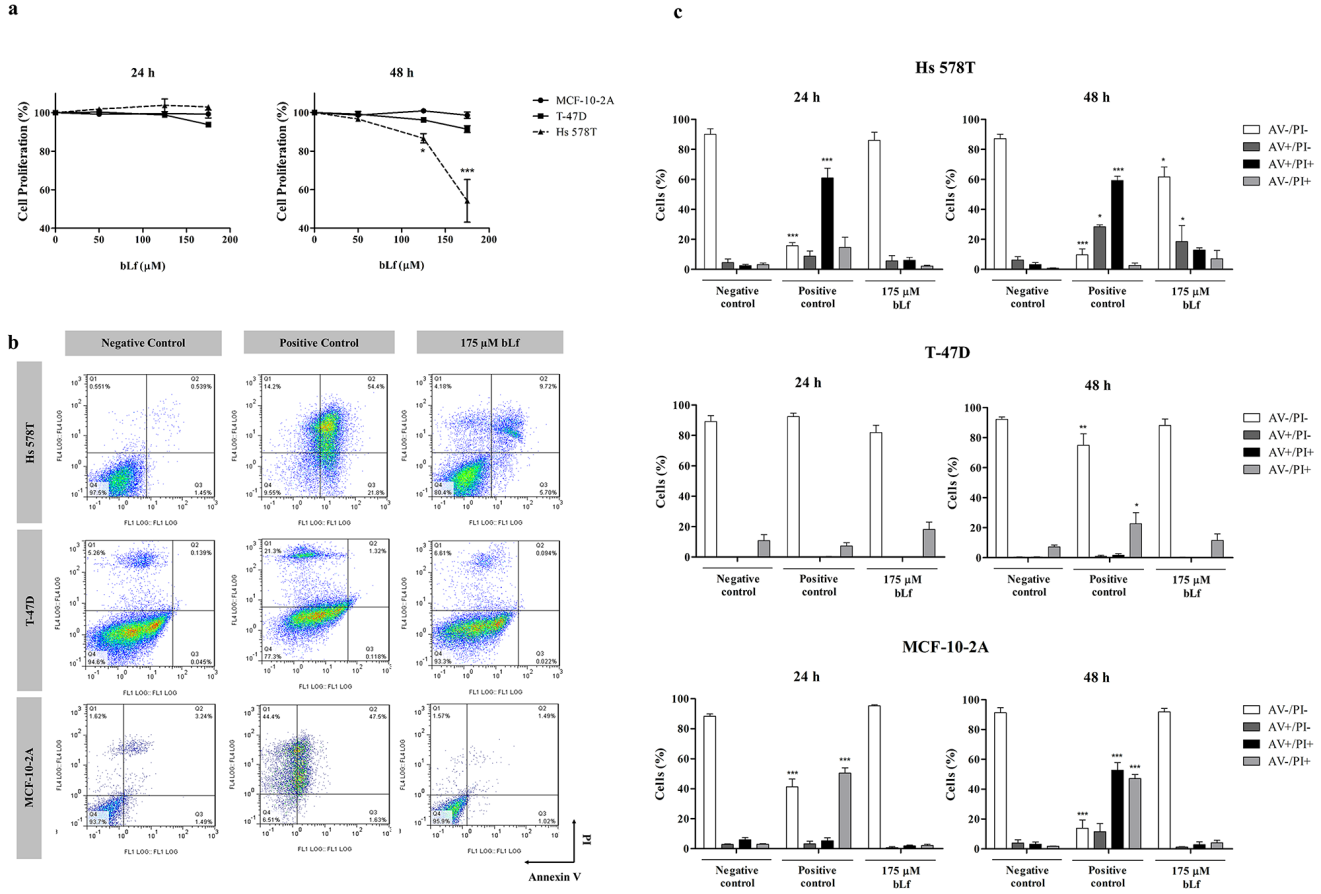
Breast cell lines display different susceptibility to bLf **a.** Analysis of cell proliferation of Hs 578T, T-47D and MCF-10-2A breast cell lines treated with increasing concentrations of bLf for 24 and 48 h. For each time point, proliferation was estimated by the SRB assay in relation to untreated cells (considered to have 100% proliferation). Values represent mean ± S.E.M. of three independent experiments, *P<0.05; ***P<0.001 compared with MCF-10-2A cells. **b.** Analysis of cell death determined by Annexin V fluorescein isothiocyanate (AV-FITC) and propidium iodide (PI) assay in Hs 578T, T-47D and MCF-10-2A cells. Representative histograms of cells double-stained with AV and PI. Cells were incubated with fresh medium in the absence (negative control) or presence of 40 μM cisplatin (positive control) or 175 μM bLf for 48 h, labelled with Annexin V and PI and analysed by flow cytometry. **c.** Quantitative analysis of AV/PI staining in the same cells incubated for 24 and 48 h in the same conditions. Values represent mean ± S.E.M. of three independent experiments, *P<0.05; ***P<0.001 when compared to negative control cells.

In order to identify the molecular targets underlying the anticancer activity of bovine lactoferrin (bLf), we analysed its effect on the proliferation of three breast cell lines with different molecular phenotypes. Hs 578T and T-47D cell lines (highly and poorly metastatic breast cancer cells, respectively), as well as the non-tumorigenic cell line MCF-10-2A (a control for normal epithelial breast cells), were incubated with 50 - 175 μM bLf for 24 and 48 h. Results showed that the proliferation of T-47D and MCF-10-2A cells, measured by the sulforhodamine B (SRB) assay, was not significantly affected after exposure to bLf for up to 48 h. However, the proliferation of the highly metastatic cell line Hs 578T exposed to 175 μM bLf decreased by 50% after 48 h of treatment (Figure 1a).

To evaluate if the observed decrease in cell proliferation was due to the induction of apoptosis by bLf, we performed the FITC-Annexin V/propidium iodide (AV/PI) assay. Results confirmed that this protein induces exposure of phosphatidylserine to the outer leaflet of the plasma membrane after 48 h of incubation only in the Hs 578T cell line (Figure 1b and 1c). Figure 1b illustrates representative biparametric histograms of cells treated with 175 μM of bLf in comparison with cisplatin-treated (positive control) and untreated cells (negative control). As it can be seen, the number of early and late apoptotic cells (AV^+^/PI^−^ and AV^+^/PI^+^) increased from 9.4% in the negative control to 31.4% and 87.7% after 48 h of incubation with 175 μM bLf and 40 μM cisplatin, respectively (Figure 1c). Moreover, the levels of necrotic cells (AV^−^/PI^+^) were very low, suggesting that bLf induces apoptosis rather than necrosis in Hs 578T cells. AV/PI staining also indicated that T-47D and MCF-10-2A cell lines were more resistant to the pro-apoptotic effect of 175 μM bLf than Hs 578T cells because, after 48 h of incubation, early and late apoptotic cells were not detected in both cell lines (Figure 1c).

**Figure 2:**
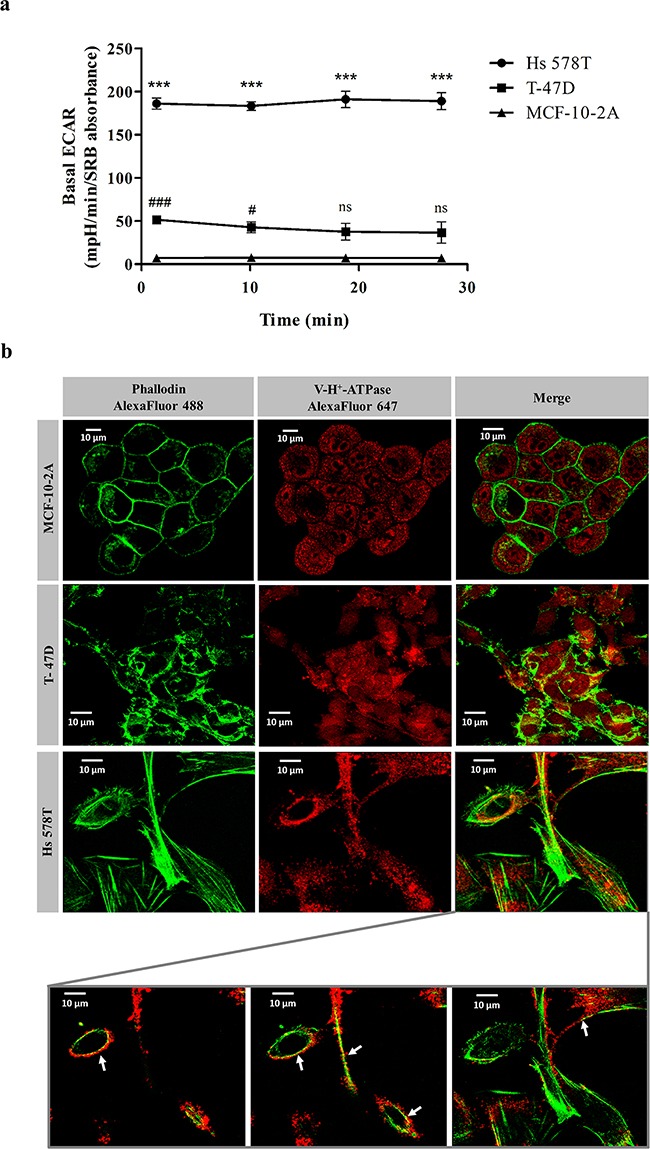
Basal extracellular acidification rate and cellular distribution of V-H+-ATPase on breast cells **a.** Real-time measurement of the basal extracellular acidification rates (ECARs) of  MCF-10-2A, T-47D and Hs 578T breast cells. Prior to ECAR measurements, cells were incubated for 1 h in assay medium as described in the Materials and Methods section. ECAR values were measured in a XF24 Seahorse Extracellular Flux Analyser over a 30 min period time during which 4 measurements were performed. Results are expressed in mpH per min per SRB absorbance at 540 nm. Values represent the mean ± S.E.M. of three independent experiments, ^ns^ non-significant; ^#^ *P<*0.05; ***/^###^ *P<*0.001 compared with MCF-10-2A cells. **b.** Representative images of immunofluorescence in the same three breast cells using confocal microscopy. Cells were incubated with primary monoclonal antibody against the *c′* subunit of V-H^+^-ATPase and secondarily labelled with Alexa fluor-647 (red fluorescence). Cytoskeleton was labelled with Alexa fluor 488-Phalloidin (green fluorescence). Insert: co-localisation of F-actin and V-H^+^-ATPase (yellow dots – arrows) observed in 3 optical sections of Hs 578T cells.

We next questioned whether the preferential cytotoxicity of bLf against the highly metastatic cancer cells could be related to the improved capacity of these cells to acidify the extracellular medium mediated by plasmalemmal V-H^+^-ATPase. To address this hypothesis, we first monitored the basal extracellular acidification rate (ECAR) of the three cell lines under study using a XF Extracellular Flux Analyser, which allows measuring the non-glycolytic acidification often named basal ECAR. Before recording the ECAR, cells cultivated for 24 h were transferred to a glucose-free medium to exhaust their glycolytic reserves and then incubated for 1 h in a non-CO_2_ incubator. This procedure enables more accurate measurements of proton extrusion, through elimination of the acidification component caused by the efflux of lactic acid. As can be observed in Figure 2a, the basal ECAR of the highly metastatic Hs 578T cells was approximately 4- and 25-fold higher than that of T-47D and MCF-10-2A cells, respectively.

Since the distinct basal ECARs suggested a different cellular localisation of V-H^+^-ATPase in the three cell lines, we next performed immunofluorescence against the *c*′ subunit of the proton pump complex. The results further confirmed that the cellular localisation patterns of V-H^+^-ATPase in the three cell lines are different (Figure 2b). Notably, in the highly metastatic Hs 578T cells it was clear that the red fluorescence from V-H^+^-ATPase co-localises with the green fluorescence of F-actin (arrows) in various optical sections. This observation corroborated a preferential localisation of this pump at the plasma membrane, which supports the observations reported above regarding the ECAR. In contrast, V-H^+^-ATPase has a predominant intracellular localisation in T-47D and MCF-10-2A cells.

Altogether, our results strongly indicate that the specificity of bLf for the highly metastatic cancer cell line is associated with the higher intrinsic basal ECAR of these cells resulting from the recruitment of the V-H^+^-ATPase to the plasma membrane.

### Bovine lactoferrin selectively inhibits the extracellular acidification rate and induces intracellular acidification in highly metastatic breast cancer cells

**Figure 3:**
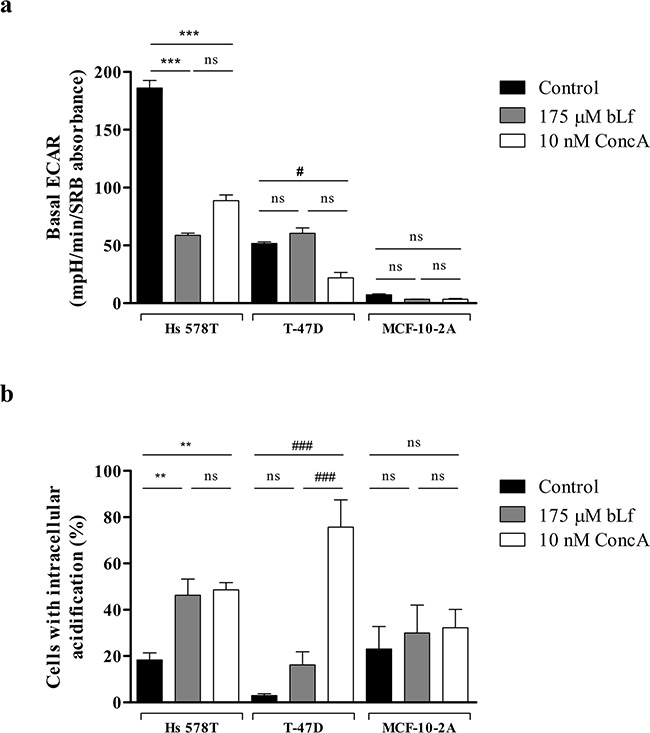
bLf inhibits the extracellular acidification rate and induces intracellular acidification in the highly metastatic Hs 578T cancer cells **a.** Real time measurement of the basal ECAR of MCF-10-2A, T-47D and Hs 578T breast cells after 24 h incubation with only culture medium, 175 μM bLf or 10 nM ConcA, as indicated. Prior to ECAR measurement in a XF24 Seahorse Extracellular Flux Analyser, cells were incubated for 1 h in assay medium as described in the Materials and Methods section. Results are expressed in mpH per min per SRB absorbance at 540 nm. **b.** Analysis of the intracellular pH in Hs 578T, T-47D and MCF-10-2A cell lines untreated or treated with 175 μM bLf or 10 nM ConcA for 24 h using the pH-sensitive probe BCECF-AM, by flow cytometry. Quantification of the percentage of cells with intracellular acidification (decreased FL1/FL4 fluorescence ratio) in comparison with the untreated cells. Values represent the mean ± S.E.M. of three independent experiments, ^ns^ non-significant; ^#^ *P<*0.05; **P<0.01; ***/^###^ *P<*0.001 compared with the control (cells without treatment) of each cell line.

To further investigate the relation between the localisation of V-H^+^-ATPase at the plasma membrane and bLf cytotoxicity suggested by the previous results, basal ECAR measurements were performed in cells incubated with either bLf or the specific V-H^+^-ATPase inhibitor concanamycin A (ConcA). For this purpose, cells were pre-incubated for 24 h in the absence of any potential inhibitor or in the presence of 175 μM bLf or 10 nM ConcA, and the basal ECAR was monitored as described above. Results showed that both bLf and ConcA inhibited the basal ECAR by more than 50% only in Hs 578T cells. Indeed, in T-47D cells, ConcA, but not bLf, was able to inhibit the basal ECAR, while in the non-tumorigenic MCF-10-2A cells none of the compounds affected the already very low basal ECAR (Figure 3a).

The pH sensitive probe BCECF-AM diffuses into cells where it is cleaved by intracellular esterases to the unesterified form, which emits fluorescence according to the intracellular pH (pHi) [27]. The effect of bLf and ConcA on the pHi was estimated using this probe and the results were expressed as the percentage of cells for which there was a measurable decrease in the ratio of green/red fluorescence intensities (FL1/FL4), indicative of intracellular acidification, when compared to the control (Supplementary Figure S1). Results are shown in Figure 3b for the three cell lines. While bLf induced intracellular acidification only in Hs 578T cells, ConcA caused intracellular acidification in both Hs 578T and T-47D cancer cell lines after 24 h of treatment. No significant changes in the intracellular pH were observed in MCF-10-2A cells after incubation with either bLf or ConcA.

Altogether, these data strongly suggest that the mechanism by which bLf and ConcA decrease the extracellular acidification rate and induce intracellular acidification is similar, involving the inhibition of V-H^+^-ATPase at the plasma membrane. This inhibitory effect of bLf may underlie its preferential pro-apoptotic effect in highly metastatic breast cancer cells and the resistance of the lowly metastatic and non-tumorigenic cells to this protein.

### The well-characterised highly metastatic breast cancer cell line MDA-MB-231 is also susceptible to bLf

**Figure 4:**
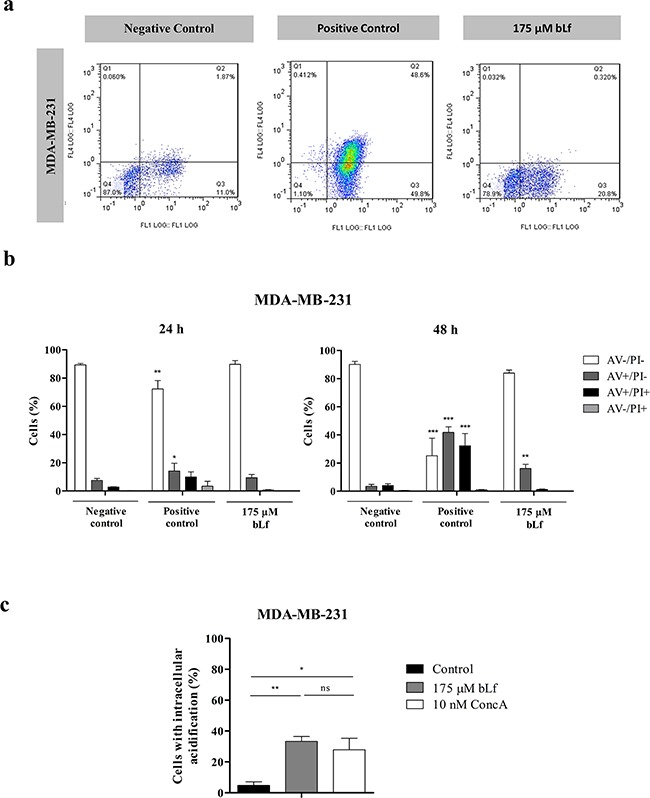
bLf induces apoptosis and intracellular acidification in the highly metastatic breast cancer cell line MDA-MB-231 **a.** Analysis of cell death determined by Annexin V/PI assay in MDA-MB-231 cells. Representative histograms of cells double-stained with AV and PI. Cells were incubated with fresh medium in the absence (negative control) or presence of 40 μM cisplatin (positive control) or 175 μM bLf for 48 h, labelled with Annexin V and PI and analysed by flow cytometry. **b.** Quantitative analysis of AV/PI staining in the same cells incubated for 24 and 48 h in the same conditions. Values represent mean ± S.E.M. of three independent experiments, *P<0.05; **P<0.01; ***P<0.001 when compared to negative control cells. **c.** Analysis of the intracellular pH in MDA-MB-231 cells untreated or treated with 175 μM bLf or 10 nM ConcA for 24 h using the pH-sensitive probe BCECF-AM, by flow cytometry. Quantification of the percentage of cells with intracellular acidification (decreased FL1/FL4 fluorescence ratio) in comparison with the untreated cells. Values represent the mean ± S.E.M. of three independent experiments, ^ns^ non-significant; * *P<*0.05; ** *P<*0.01; compared with the control (cells without treatment).

It has been previously demonstrated that V-H^+^-ATPase is recruited to the cell surface in the highly metastatic breast cancer cell line MDA-MB-231, much like we observed in the present study in the case of the Hs 578T cells. Furthermore, it was found that this proton pump displays higher biochemical activity at the plasma membrane of highly metastatic than poorly metastatic breast cancer cell lines [7]. To investigate if the selective cytotoxicity induced by bLf in the Hs 578T cells was specific for this cell line or could be extended to other highly metastatic breast cancer cells, we studied its effect also on the MDA-MB-231 cell line. For that purpose we tested the effect of bLf in apoptosis induction and in the intracellular acidification in these cells. As shown in Figures 4a and 4b, the AV/PI assay revealed that, likewise, bLf induces apoptosis in these cells, because the percentage of early and late apoptotic cells (AV^+^/PI^−^ and AV^+^/PI^+^) increased from 7.6% in the negative control to 17.4% and 74% in the bLf- and cisplatin-treated cells, respectively. Similarly to what we observed in the Hs 578T cells, both bLf and ConcA induced intracellular acidification in MDA-MB-231 cells measured with the BCECF-AM probe (Figure 4c).

### Bovine lactoferrin inhibits both the proton pumping and hydrolytic activities of V-H^+^-ATPase

**Figure 5:**
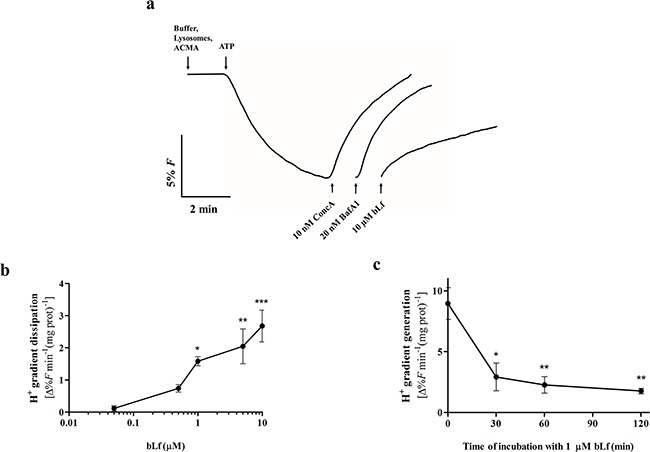
bLf, like ConcA and BafA1, inhibits V-H^+^-ATPase proton pumping activity in purified rat liver lysosomes **a.** Typical fluorescence signal of the initial velocity of proton pumping by V-H^+^-ATPase in a lysosomal suspension after adding 0.5 mM ATP, and immediate dissipation of the proton gradient by addition of 10 μM bLf, 10 nM ConcA and 20 nM BafA1. **b.** Quantification of the immediate effect of increasing concentrations of bLf on the proton gradient dissipation. Values are expressed as the fluorescence percentage variation of the proton gradient dissipation per min per mg protein. *P<0.05; **P<0.01; ***P<0.001 in comparison with the control. **c.** Time course inhibition of the initial velocity of the V-H^+^-ATPase proton pumping activity along incubation with 1 μM bLf for 120 min. Values are expressed as the fluorescence percentage variation of the proton gradient generation per min per mg protein. *P<0.05; **P<0.01compared with time zero of incubation.

Proton pumping activity of V-H^+^-ATPase was monitored spectrofluorimetrically in purified rat liver lysosomes by measuring the fluorescence quenching of the pH-sensitive dye ACMA after addition of ATP. In this well-established experimental system [28], we were able to perform a wealth of experiments to measure V-H^+^-ATPase activity, including kinetic and inhibition studies due to the high amount of membrane protein obtained, and to the high purity of the isolated fraction. Indeed, our lysosomal fraction isolated from rat liver showed a reduced cytosolic and mitochondrial contamination (Supplementary Figure S2), thus minimizing possible interference of the mitochondrial F-H^+^-ATPase in the V-H^+^-ATPase activity measurements. As seen in Figure 5a, bLf inhibited the proton pumping activity of lysosomal V-H^+^-ATPase when added at the steady state, although to a lower extent than 10 nM ConcA and 20 nM BafA1. At concentrations above 1 μM, bLf dissipated the proton gradient generated by the addition of ATP in a dose-dependent manner (Figure 5b). Furthermore, incubation of lysosomes with 1 μM bLf for 30 min was sufficient to reduce the initial velocities of proton pumping activity upon addition of ATP by approximately 65%, and longer incubation times did not increase this inhibitory effect (Figure 5c). We further confirmed that the V-H^+^-ATPase from cancer cell lines is equally inhibited by bLf when proton pumping activity studies were performed in isolated crude membrane fractions (Supplementary Figure S3). The difference in the concentration range of bLf and BafA1/ConcA needed to inhibit V-H^+^-ATPase is likely due to their different chemical nature and consequent level of inhibition. In fact, bLf is a globular protein with a molecular weight of 80 kDa and BafA1/ConcA are macrolide antibiotics with a molecular weight of 622 Da and 866 Da, respectively.

**Figure 6:**
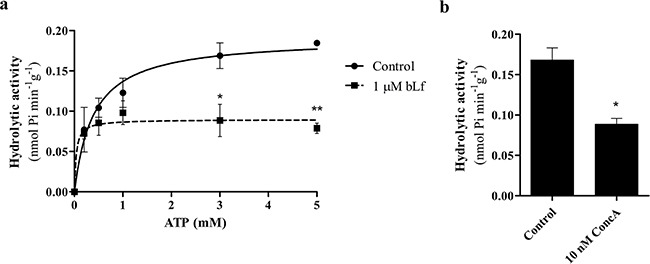
bLf inhibits V-H^+^-ATPase hydrolytic activity in purified rat liver lysosomes **a.** ATP-dependent V-H^+^-ATPase hydrolytic activity kinetics of rat liver lysosome suspensions. The continuous line is derived by fitting the data points to a Michäelis–Menten kinetics and the corresponding kinetic parameters are: *K*_m_, 0.40 ± 0.11 mM ATP and *V*_max_, 0.19 ± 0.01 nmol^−1^min^−1^g protein (mean ± SD). The V-H^+^-ATPase hydrolytic activity was also measured with increasing concentrations of ATP in the presence of 1 μM bLf. With 3 and 5 mM ATP, bLf significantly inhibits V-H^+^-ATPase hydrolytic activity. **b.** V-H^+^-ATPase hydrolytic activity was measured in control lysosomes and incubated with 10 nM ConcA for 30 min at 37°C with 3 mM ATP. *P<0.05; **P<0.01compared with the hydrolytic activity measured in control lysosomes.

We next evaluated the effect of bLf on the hydrolytic activity of V-H^+^-ATPase from rat liver lysosomes (Figure 6). The rate of ATP hydrolysis in the lysosome suspensions, in the absence of any potential inhibitor, followed a simple Michäelis-Menten kinetics, in agreement with previous studies [29, 30], and the corresponding kinetic parameters were as follows: *K*_m_, 0.40 ± 0.11 mM ATP and *V*_max_, 0.19 ± 0.01 nmol^−1^min^−1^g protein. At saturating ATP concentrations (0.3 and 0.5 mM), the presence of 1 μM bLf inhibited the initial rates of ATP hydrolysis by approximately 50% (Figure 6a), like 10 nM ConcA (Figure 6b). Similar inhibitory effects on V-H^+^-ATPase hydrolytic activity by ConcA were previously reported [31]. Overall, our results demonstrate that bLf inhibits the V-H^+^-ATPase proton pumping activity in a concentration-dependent manner, as well as its hydrolytic activity.

## DISCUSSION

The concept of “magic bullet” was proposed by Paul Ehrlich in 1906 to describe the ideal therapeutic agent that would exert its full action exclusively on its targets, without side-effects. In chemotherapy, it represents a drug that selectively targets cancer cells of a patient without harming healthy cells [32]. Can the naturally occurring Lf be considered a “magic bullet”?

In this work, we showed that the highly metastatic breast cancer cell lines Hs 578T and MDA-MB-231 are more susceptible to bLf than the poorly metastatic T-47D and the non-tumorigenic MCF-10-2A breast cell lines. In addition, we showed that Hs 578T cells exhibit a higher intrinsic extracellular acidification rate (ECAR) under basal conditions that is associated to a prominent localisation of V-H^+^-ATPase at the plasma membrane, as previously described for the MDA-MB-231 cell line [7]. Furthermore, bLf decreases the basal ECAR by almost 70% in Hs 578T cells and causes intracellular acidification in both Hs 578T and MDA-MB-231 cells. These results strongly suggest that V-H^+^-ATPase inhibition accounts for the selectivity of bLf for highly metastatic cancer cells. Accordingly, the absence of V-H^+^-ATPase at the plasma membrane associated with the lower basal ECAR in the poorly metastatic T-47D and non-tumorigenic MCF-10-2A cells, endows these two cell lines a much lower sensitivity to bLf. This newly identified bLf mechanism of action, together with the peculiar features of the highly metastatic cancer cells reveal that this natural protein may own the desirable features of a magic bullet, at least against this type of cancer cells. Interestingly, the results of finished clinical trials are in good agreement with our data as they demonstrated Lf treatment efficacy against metastatic cancers, and attested its safety and tolerability, with no serious adverse effects identified [33, 34]. In fact, orally administered recombinant human Lf (rhLf) delayed tumour progression in patients with metastatic non-small cell lung cancer or renal cell carcinoma, with no significant hematologic, hepatic, or renal toxicities reported.

Previous studies showed that the cytotoxicity of Lf depends on its purity and iron saturation level, as well as on the cell type. In fact, depending on the cellular context, iron-free and iron-saturated forms of Lf have different effects being either one more effective than the other [24] or, in some cases, having opposite effects [35]. In this study, we found that incubation for 48 h with 50 to 175 μM of native bLf, exhibiting 21% iron saturation, inhibited the proliferation of the metastatic breast cancer cell line Hs 578T, which was associated with a strong perturbation of extra- and intracellular pH and apoptosis. In parallel, an immediate inhibition of V-H^+^-ATPase mediated-proton pumping by up to 10 μM bLf was observed in purified systems like crude membrane fractions and isolated lysosomes.

The main outcome of our study is the finding that bLf acts as a V-H^+^-ATPase inhibitor. Similarly to bLf, the enhanced anticancer activity of the V-H^+^-ATPase inhibitors against metastatic cancer cell lines was also demonstrated [7, 36]. Moreover, their effectiveness was shown in preclinical models of cancer metastasis [11, 37, 38]. Therefore, these compounds exhibit good potential to be used in therapeutic strategies for metastatic human cancers. However, their use in clinic has fallen short of expectations mainly because of their cytotoxicity and lack of understanding of their mechanism of action [8]. On the other hand, it is generally recognised that natural products have particular advantages over conventional anticancer drugs, including reduced toxicity and side effects, promotion of the natural immune system and reduced risk of patient drug resistance [39]. In the light of our results and of the advantages of using natural compounds, bLf represents therefore an interesting avenue for future research in this field. Moreover, several other advantages such as the availability of bLf from natural sources, its already established large-scale production, its easy and safe oral administration, as well as its beneficial effects in health promotion and treatment of diseases including cancer [40], make bLf a much more attractive compound than the classical V-H^+^-ATPase inhibitors available so far.

**Figure 7:**
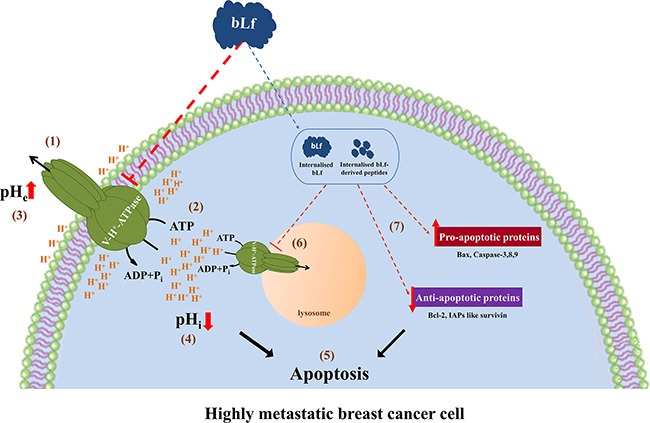
Working model for the molecular mechanism underlying the pro-apoptotic anticancer activity of bLf The exposure of highly metastatic breast cancer cells to bLf leads to the inhibition of plasmalemmal V-H^+^-ATPase proton pumping (1) and hydrolytic (2) activities. The inhibition of proton efflux and the ensuing extracellular alkalinisation (3) and intracellular acidification (4) triggers apoptosis (5). bLf and/or its derived peptides can also be internalised to inhibit the lysosomal V-H^+^-ATPase (6) and modulate the levels of key apoptotic proteins (7), thus amplifying the apoptotic process (5).

Since bLf has an immediate inhibitory effect on V-H^+^-ATPase activity in isolated lysosomes, it appears that it acts on plasmalemmal V-H^+^-ATPase, and the observed recruitment of this proton pump to the plasma membrane in highly metastatic cancer cells explains their high susceptibility to bLf. The inhibition of the plasmalemmal V-H^+^-ATPase likely conveys from the cell surface to the intracellular “milieu” an early cytosolic acidification, which subsequently may trigger apoptosis (Figure 7). Extracellular acidity is one of the main contributing factors for cancer's multidrug resistance, through protonation and neutralization of chemotherapeutics outside the cell [9]. Therefore, the effect of bLf on the basal ECAR, and hence on the extracellular pH also provides the possibility to combine bLf with other anti-neoplastic drugs to design effective anti-breast cancer strategies. Indeed, Lf was demonstrated to augment the chemotherapeutic effects of tamoxifen in a mouse model of metastatic 4T1 breast cancer [41].

Future studies on the molecular interactions between bLf and V-H^+^-ATPase may reveal new mechanistic insights on the inhibition of V-H^+^-ATPase by this protein. According to our working model, bLf may have short and long-term effects (Figure 7). We hypothesise that, in the short-term bLf directly or indirectly interacts with V-H^+^-ATPase at the plasma membrane inhibiting its activity. In any case, V-H^+^-ATPase inhibition results in ECAR decrease and subsequent intracellular acidification that triggers apoptosis. In addition, bLf and/or its derived peptides may be internalised and cause long-term effects after reaching their intracellular targets, like the lysosomal V-H^+^-ATPase, thus amplifying the apoptotic process. This mechanism may contribute to the already described differential increase of apoptosis by Lf through upregulation/downregulation of the pro/anti-apoptotic proteins and caspase activation [12, 16, 17, 20]. Thus, the set-up of preclinical studies aiming to explore the effects of bLf on the TME acidity and TME-dependent metastasis warrants further attention.

In summary, we found that, in highly metastatic breast cancer cells, bLf inhibits V-H^+^-ATPase, a recognised molecular target for anti-breast cancer therapy. We propose that bLf behaves as a V-H^+^-ATPase inhibitor and diminishes the TME acidity by inhibiting plasmalemmal V-H^+^-ATPase. It therefore acts on one of the “cancer's Achilles heels” [42] and hence can prevent tumour growth and metastasis. Thus, our findings may lay the foundation for future *in vivo* studies aiming to exploit the use of this natural protein as a novel V-H^+^-ATPase inhibitor for the treatment of highly metastatic breast cancers, seemingly with advantages over the V-H^+^-ATPase inhibitors currently available. As a personalised cancer therapy is desirable, detection of V-H^+^-ATPase at the plasma membrane of breast cancer cell biopsies by immunohistochemistry could indicate whether bLf should be used as part of the therapeutic strategy.

## MATERIALS AND METHODS

### Reagents and antibodies

Bovine lactoferrin (bLf) was obtained from DMV (Veghel, The Netherlands). According to the manufacturer, the protein is approximately 80% pure, with 3.5% moisture, and 21% iron-saturated. bLf was dissolved in phosphate buffered saline (PBS, 1.37 M NaCl, 2.7 mM KCl, 10 mM Na_2_HPO_4_, 1.8 mM KH_2_PO_4_, pH 7.4) to obtain the different concentrations used throughout this work.

Concanamycin A (ConcA), sulforhodamine B (SRB), cisplatin [cis-diamineplatinum(II) dichloride] and paraformaldehyde (PFA) were obtained from Sigma-Aldrich. Bafilomycin A1 (BafA1) was purchased from Acros Organics. ACMA (9-amino-6-chloro-2-methoxyacridine), Alexa fluor 488-Phalloidin and Alexa fluor 647 were obtained from Molecular Probes. FITC Annexin V apoptosis detection kit was purchased from BD Bioscience. Vectashield mounting medium was purchased from Vector Laboratories. The anti-V-H^+^-ATPase *c′* subunit antibody was purchased from Millipore-Merck, anti-Tom20 from Santa Cruz Biotechnology, and anti-GAPDH from Hytest; the secondary antibodies Peroxidase-AffiniPure goat anti-rabbit IgG and goat anti-mouse IgG were acquired from Jackson ImmunoResearch.

### Cell lines and culture conditions

Human breast cancer cell lines T-47D (HTB-113; ATCC), Hs 578T (HTB-126; ATCC) and MDA-MB-231 (HTB-26; ATCC) were grown in Dulbecco's modified Eagle's medium (DMEM) supplemented with 10% fetal bovine serum and 1% penicillin/streptomycin. MCF-10-2A (CRL-10781; ATCC) cells were grown in DMEM-F12 medium supplemented with 5% horse serum, 1% penicillin/streptomycin, 20 ng/ml epidermal growth factor (EGF), 100 ng/ml cholera toxin, 0.01 mg/ml insulin and 500 ng/ml hydrocortisone. All cell lines were maintained at 37°C in a humidified atmosphere with 5% CO_2_. For all experiments, cells were seeded in 6-well at a concentration of 1.5×10^5^ cells/ml for 24 h experiments and 1×10^5^ cells/ml for 48 h experiments, with the exception of the ECAR experiments in which cells were seeded in XF 24-well plates at a concentration of 1×10^4^ cells/well. All medium constituents were acquired from Biochrom with the exception of EGF, cholera toxin, hydrocortisone and insulin that were purchased from Sigma-Aldrich.

### Assessment of cell proliferation by Sulforhodamine B assay

Breast cells were seeded in 6-well plates and incubated with different bLf concentrations (50, 125 and 175 μM) for 24 and 48 h. Afterwards, cells were fixed for 90 min at −20°C in ice-cold 1% acetic acid in methanol, and then incubated with 0.5% (w/v) SRB in 1% acetic acid for 90 min at 37°C. After washing with 1% acetic acid and drying, protein-bound sulforhodamine B (SRB) was dissolved in 10 mM Tris for 10 min at room temperature (RT). A sample from each condition was transferred to a 96-well plate and absorbance was read at 540 nm in a microplate reader (SpectraMax 340PC, Molecular Devices). Results were normalized to the untreated cells, which were considered to have 100% cell proliferation.

### Annexin V/PI assay

Cells were seeded in 6-well plates and incubated for 24 and 48 h in the presence of only medium (negative control), 40 μM cisplatin (positive control) or 175 μM bLf. Apoptosis was detected using the “FITC Annexin V apoptosis detection kit” according to the manufacture instructions (BD Biosciences). Briefly, after 24 or 48 h, cells were collected and washed with PBS 1×. 2×10^5^ cells were ressuspended in 100 μL of 1× “Binding Buffer” and incubated with 1 μL AV-FITC and 1 μL PI for 15 min in the dark. Apoptosis was assessed by flow cytometry.

### Extracellular acidification rate (ECAR) measurement

Basal extracellular acidification rates of Hs 578T, T-47D and MCF-10-2A cell lines were determined using a Seahorse Extracellular Flux (XF-24) Analyser (Seahorse Bioscience). Cells were seeded into XF24 cell culture microplates at a cellular density of 10000 cells/well in their normal growth media and left to adhere overnight in a humidified 37°C incubator with 5% CO_2_. Cells were then treated with either 175 μM bLf or 10 nM ConcA for 24 h. In the wells corresponding to the negative control, the medium was changed and no treatment was added. Prior to the basal ECAR measurement, the growth medium was exchanged to a base assay medium (DMEM 5030 – Sigma-Aldrich) supplemented with 4 mM glutamine and rigorously adjusted to pH 7.35±0.05, and the plate was incubated for 1 h in a 37°C/non-CO_2_ incubator to deplete all the glycolytic reserves. Because each cell line has a different proliferation rate during the incubation period, the amount of protein present in each well was estimated by SRB assay following ECAR measurements. ECAR values were normalized to the SRB absorbance of each well using the Wave 2.2.0 software, and plotted as the mean +/− SEM, each point representing the average of three different wells.

### Intracellular pH measurement

Measurement of intracellular pH (pHi) was performed with the pH-sensitive probe BCECF-AM. Cells were seeded in 6-well plates and incubated with only medium, with 175 μM bLf or with 10 nM ConcA for 24 h. At the end of the treatment, cells were collected by tripsinisation and washed twice with HBSS 1× (53.3 mM KCl, 4.4 mM KH_2_PO_4_, 1379.9 mM NaCl, 55.5 mM Glucose, 3.3 mM Na_2_HPO_4_). Then, 2×10^5^ cells from each condition were loaded with 1 μM BCECF-AM for 30 min at 37°C. After centrifugation at 2000 rpm during 5 min to remove the medium, cells were ressuspended in HBSS 1× and analysed by flow cytometry. The percentage of cells with intracellular acidification was estimated from the decrease in the ratio of green/red fluorescence intensities (FL1/FL4) when compared to the negative control.

### Flow cytometry

Flow cytometry analysis was performed with an Epics® XL^TM^ (Beckman Coulter) flow cytometer equipped with an argon-ion laser emitting a 488 nm beam at 15 mW. Green fluorescence was collected through a 488 nm blocking filter, a 550 nm long-pass dichroic and a 525 nm band-pass filter. Red fluorescence was collected through a 560 nm short-pass dichroic, a 640 nm long-pass, and another 670 nm long-pass filter. For each sample, 20 000 events were evaluated. Data were analysed using the FlowJo software (version 7.6).

### Immunofluorescence and confocal microscopy

For immunofluorescence experiments, cells were seeded in 6-well plates containing glass coverslips. After 24 h, cells were fixed with 4% paraformaldehyde (PFA) for 40 min. After rinsing with 1× PBS, cells were incubated with 50 mM ammonium chloride for 10 min, rinsed again and permeabilised with PBS-0.1% SDS for 10 min. Cells were then blocked in PBS-3% BSA for 20 min and washed. Following this procedure, cells were incubated with anti-ductin primary antibody, which detects the *c′* subunit of V-H^+^-ATPase, in PBS-0.1% BSA overnight at 4°C in a humidified chamber. Subsequently, cells were washed with PBS-0.1% BSA and then labelled with Alexa Fluor 647 for 1 h in the dark. After rinsing, cells were incubated with Alexa Fluor 488-Phalloidin to stain F-actin, and delineate the cell cytoskeleton, for 1 h in the dark. After mounting the coverslip in Vectashield mounting medium, samples were maintained at −20°C until visualization. Images were acquired in a sequential mode by a confocal scanning laser microscope (BX61/FLUOVIEW1000, Olympus), using a 60× oil immersion objective and the specific filter settings for Alexa Fluor 488 and 647. For all the images acquired, a negative control corresponding to cells labelled only with the secondary antibody Alexa Fluor 647 (Supplementary Figure S4) was performed in order to confirm the specific staining of the V-H^+^-ATPase *c*′ subunit when the primary antibody was used.

### Isolation of rat liver lysosomes

The animal organs used in this work were from rats included in research projects approved by the Animal Ethics Committee of the Institution where the studies were performed and by the national competent authority for animal protection Direção Geral de Alimentação e Veterinária (DGAV). This study was carried out in strict accordance with the recommendations of the Guide for the Care and Use of Laboratory Animals, National Academy of Science, and the EU Directive 2010/63/EU. All personnel involved in the procedures are approved as competent for animal experimentation by DGAV.

Sprague-Dawley female rats, 24 weeks (Charles-River, Spain) were maintained in SPF-like conditions, in a humidity and temperature-controlled room (22-23°C) and on a 12 h light: 12 h dark cycle. Autoclaved water and irradiated food (4RF25-GLP, Mucedola, Settimo Milanese, Italy) were provided in an *ad libitum* regime.

The rats were euthanized with an intraperitoneal injection of penthobabital (150mg/kg) (Eutasil, Ceva Saúde Animal, Portugal) and their livers collected and immediately stored in a cold buffer (0.25 M sucrose in 10 mM Tris-HCl, pH 7.4). Then, the liver was minced and homogenized in a Potter-Elvehjem. After centrifugation at 12000 g for 10 min, the pellet was discarded and a CaCl_2_ solution (0.08 M CaCl_2_ in 0.25 M sucrose in 10 mM Tris-HCl, pH 7.4) was added to the supernatant at a final concentration of 8 mM to cause mitochondrial swelling. The sample was then centrifuged at 25000 g for 15 min. The pellet was resuspended in 30 mL of KCl buffer (150 mM KCl in 10 mM Tris-HCl, pH 7.4) and centrifuged again at 25000 g for 15 min. The resulting pellet was finally resuspended in 2 mL of KCl buffer and kept on ice.

### Protein quantification

Protein concentration was determined by the Lowry method [43], using BSA (5 μg/μL - 25 μg/μL) as a standard.

### Measurement of V-H^+^-ATPase proton pumping activity

Proton pumping activity of V-H^+^-ATPase in isolated lysosomes/crude membranes was evaluated by measuring ACMA fluorescence quenching in a spectrofluorimeter (LS-5B, Perkin-Elmer) [44]. The excitation/emission wavelengths were set to 415 nm and 485 nm, respectively. The reaction medium contained 1 mM MOPS (3-(N-morpholino)propanesulfonic acid)-Tris pH 7.2, 100 mM KCl, 2 μM ACMA, 12.5 mM MgCl_2_, and 200 μg of protein of lysosome in a final volume of 2 ml. The reaction was initiated by adding 0.5 mM ATP and the rate of initial fluorescence quenching was recorded. 10 nM ConcA, 20 nM BafA1 and different concentrations of bLf (0.05, 0.5, 1, 5, 10 μM) were added to the assay medium at steady-state to study their inhibitory effects. To study the effect of the incubation time on the V-H^+^-ATPase H^+^-pumping activity, lysosomes were pre-incubated with 1 μM bLf for 30, 60 and 120 min, before ATP addition. The ACMA fluorescence quenching was considered as the V-H^+^-ATPase proton transport activity [Δ%*F* min^−1^ (mg prot)^−1^], and the fluorescence quenching recovery as the inhibition of this activity. The results were analysed using the GraphPad Prism Software.

### Measurement of V-H^+^-ATPase hydrolytic activity

The rate of ATP hydrolysis was determined by measuring the release of inorganic phosphate (Pi) according to a procedure described elsewhere [45] with modifications. Isolated lysosomes (50 μg protein) were mixed with 300 μL of 3 mM ATP (or different ATP concentrations for the kinetic analysis), 0.02% Triton X-100, 50 mM KCl, 1 mM sodium molybdate, 6 mM MgSO_4_ in 30 mM Tris pH 8, with or without additional test compounds, and incubated for 30 min at 37°C, with slow agitation (80 rpm). The reaction was stopped by adding 500 μL of a cold solution containing 10% trichloroacetic acid (TCA) and 4% perchloric acid. Samples were kept on ice for 5 min, centrifuged for 3 min at 2400 g, and 500 μL of the supernatant were mixed with 1.3 mL of Ames solution composed of 1 volume of 10% ascorbic acid mixed with 6 volumes of 21.4 mM ammonium molybdate and 53.6 mM of H_2_SO_4_. After 15 min at RT in the dark, absorbance was read at 820 nm using a blank control performed without protein and NaH_2_PO_4_ as standard to establish a calibration curve. For all the experiments, a control sample without protein was prepared to subtract the spontaneous hydrolysis induced by the compounds. The V_max_ and *K*_m_ values of the ATP hydrolysis rate were estimated by fitting the data points to a Michäelis-Menten type kinetics with the GraphPad Prism Software.

### Western blot analysis

Protein samples (50 μg) of isolated lysosomal fractions or cell homogenates were separated by sodium dodecyl sulfate 15% polyacrylamide gel electrophoresis and transferred onto PVDF (polyvinylidene difluoride, Millipore-Merck) membranes. Next, membranes were blocked in 5% non-fat milk in PBS-0.1% Tween 20 for 2 h, with agitation to avoid non-specific interactions. Membranes were then incubated overnight at 4°C with the primary antibodies, namely rabbit polyclonal anti-V-H^+^-ATPase *c′* subunit (Millipore-Merk), rabbit polyclonal anti-Tom20 (Santa Cruz Biotechnology) and mouse monoclonal anti-GAPDH (Hytest), followed by incubation with secondary antibodies Peroxidase-AffiniPure goat anti-rabbit IgG or goat anti-mouse IgG (1:2000; Jackson ImmunoResearch). Chemiluminescence detection was performed using the ECL detection system (Millipore-Merck) and a Chemi-Doc XRS system (BioRad).

### Statistical analysis

Data are expressed as means ± S.E.M. of at least three independent experiments. Statistical analysis was performed using one-way ANOVA followed by Bonferroni post-test using GraphPad Prism, version 5.0.

## SUPPLEMENTARY FIGURES


