# Supplementary material for: Lactoferrin selectively triggers apoptosis in highly metastatic breast cancer cells through inhibition of plasmalemmal V-H+-ATPase
Source: Oncotarget. 2016 Aug 19;7(38):62144–58. doi: 10.18632/oncotarget.11394 (PMC5308717; doi:10.18632/oncotarget.11394)
Supplement: Supplementary file 1 [file oncotarget-07-62144-s001.pdf]

Lactoferrin selectively triggers apoptosis in highly metastatic breast cancer cells through inhibition of plasmalemmal V-H$^+$-ATPase

SUPPLEMENTARY FIGURES

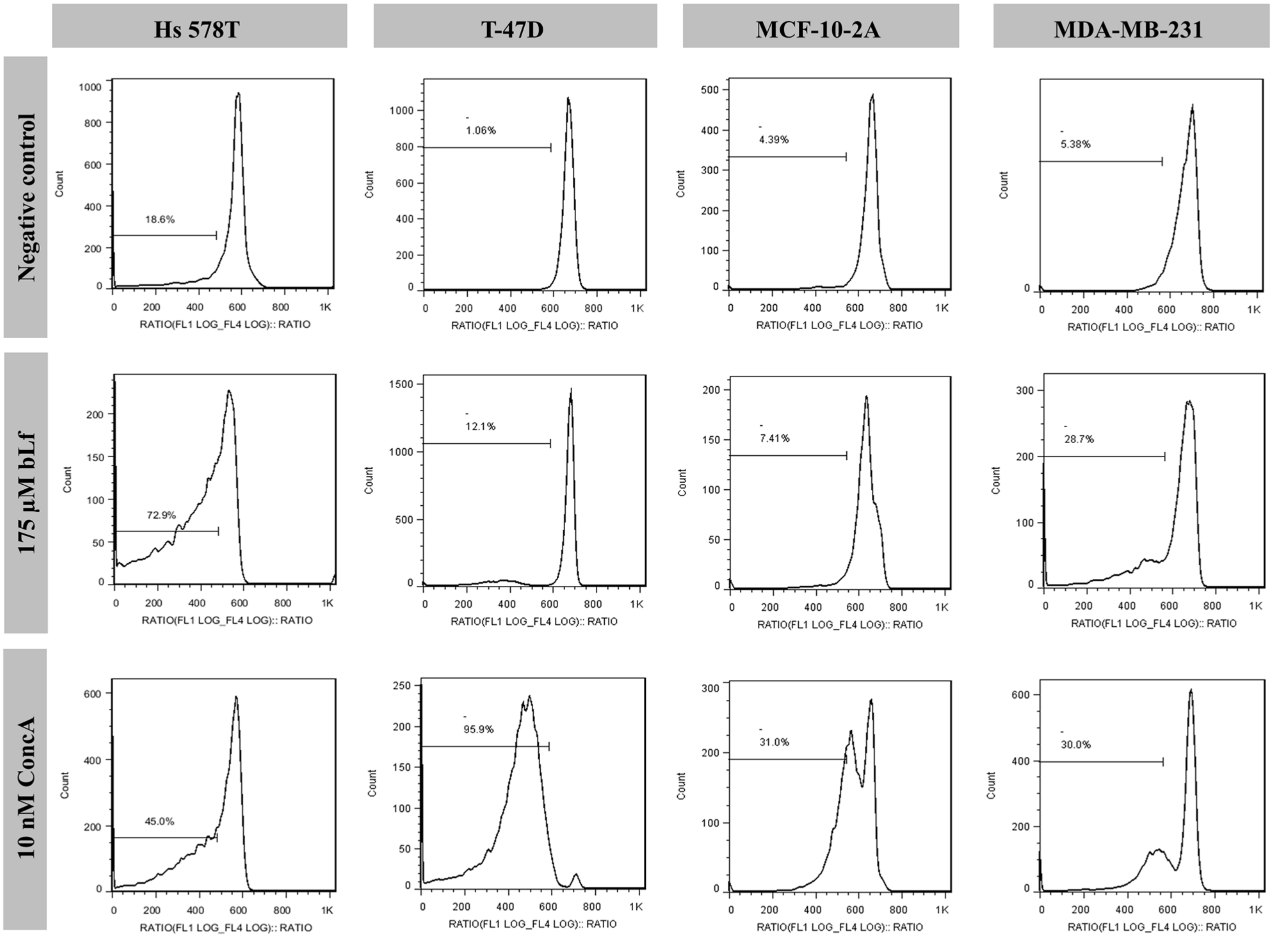

Supplementary Figure S1: Analysis of the bLf-induced alterations in intracellular pH. Representative histograms of the analysis of the intracellular pH in Hs 578T, T-47D, MCF-10-2A and MDA-MB-231 cell lines untreated or treated with 175 µM bLf or 10 nM ConcA for 24 h using the pH-sensitive probe BCECF-AM, by flow cytometry. The x-axis represents the ratio between green and red fluorescence intensities (FL1/FL4). Percentage of cells with intracellular acidification (lower BCECF fluorescence) is indicated for each condition.

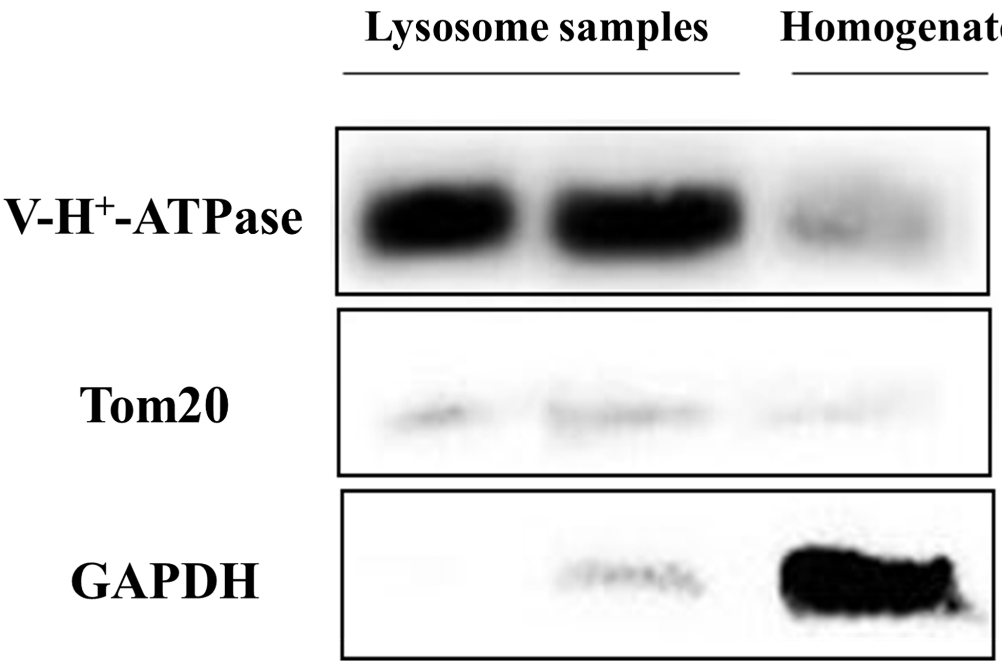

Supplementary Figure S2: Purity of isolated rat liver lysosomes subcellular fraction monitored by western blot. Western blot analysis performed in a representative cell homogenate after mitochondria sedimentation and in two representative isolated lysosome fractions (50 µg protein/lane). Samples were labelled with antibodies specific for V-H$^+$-ATPase, used as a specific lysosomal marker; Tom20, a mitochondrial marker; and GAPDH, a cytosolic protein.

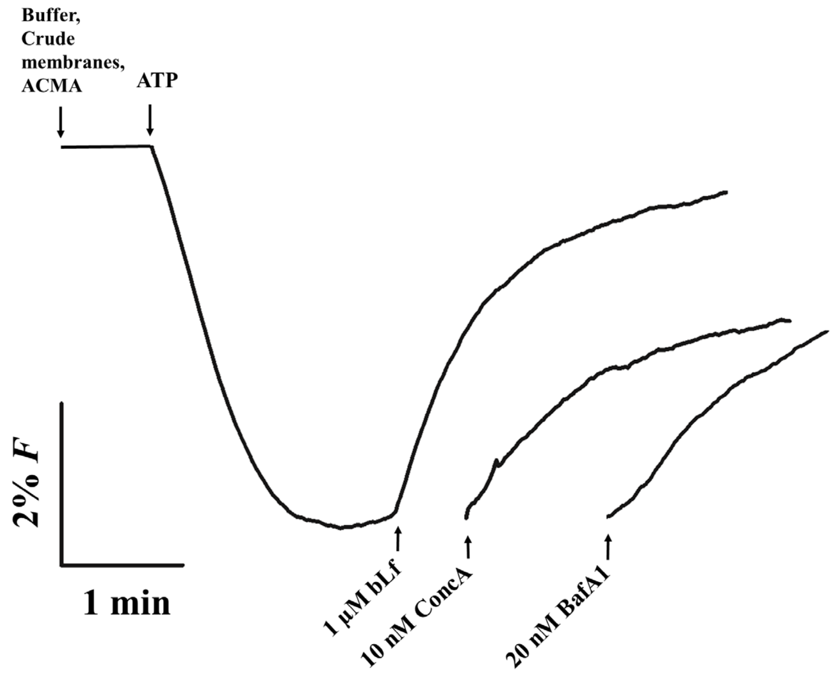

Supplementary Figure S3: bLf, like ConcA and BafA1, inhibits V-H$^+$-ATPase proton pumping activity in crude membrane fractions isolated from a cancer cell line. Typical fluorescence signal of the initial velocity of proton pumping by V-H$^+$-ATPase in a crude membrane suspension isolated from the T-47D cell line after adding 0.5 mM ATP and immediate dissipation of the proton gradient by addition of 1 µM bLf, 10 nM ConcA and 20 nM BafA1. Crude membrane fractions were obtained with the Focus SubCell kit from GBiosciences. Briefly, after harvesting and lysing the cells (5×10^7 cells/ml), a series of differential centrifugations were performed to remove nuclear, mitochondrial and cytosolic fractions, thus obtaining a fraction enriched in cellular membranes.

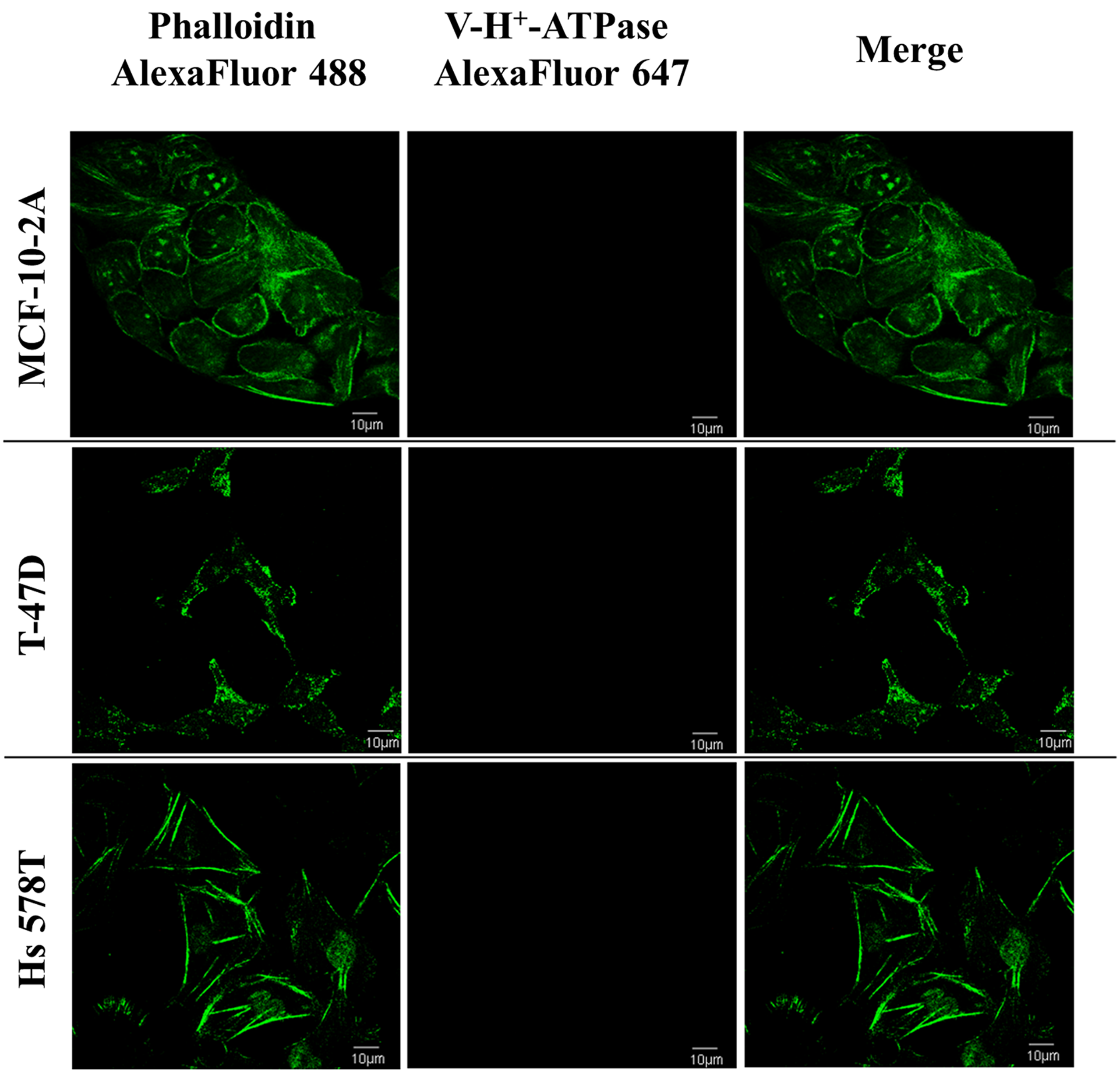

Supplementary Figure S4: Negative controls of immunofluorescence experiments. Panels correspond to the immunofluorescence negative controls in which the breast cells were incubated only with the secondary antibody Alexa fluor-647, thus demonstrating that in Figure 2b the red labelling corresponds specifically to V-H^+-ATPase subunit *c'*. Cytoskeleton was labelled with Alexa fluor 488-Phalloidin (green fluorescence).
